# Comparative Performance of Creatinine-Based Estimated Glomerular Filtration Rate Equations in the Malays: A Pilot Study in Tertiary Hospital in Malaysia

**DOI:** 10.1155/2017/2901581

**Published:** 2017-06-18

**Authors:** Maisarah Jalalonmuhali, Ng Kok Peng, Lim Soo Kun

**Affiliations:** University Malaya Medical Centre, 59100 Kuala Lumpur, Malaysia

## Abstract

**Aim:**

To validate the accuracy of estimated glomerular filtration rate (eGFR) equations in Malay population attending our hospital in comparison with radiolabeled measured GFR.

**Methods:**

A cross-sectional study recruiting volunteered patients in the outpatient setting. Chromium EDTA (51Cr-EDTA) was used as measured GFR. The predictive capabilities of Cockcroft-Gault equation corrected for body surface area (CGBSA), four-variable Modification of Diet in Renal Disease (4-MDRD), and Chronic Kidney Disease Epidemiology Collaboration (CKD-EPI) equations were calculated.

**Results:**

A total of 51 subjects were recruited with mean measured GFR 42.04 (17.70–111.10) ml/min/1.73 m^2^. Estimated GFR based on CGBSA, 4-MDRD, and CKD-EPI were 40.47 (16.52–115.52), 35.90 (14.00–98.00), and 37.24 (14.00–121.00), respectively. Higher accuracy was noted in 4-MDRD equations throughout all GFR groups except for subgroup of GFR ≥ 60 ml/min/1.73 m^2^ where CGBSA was better.

**Conclusions:**

The 4-MDRD equation seems to perform better in estimating GFR in Malay CKD patients generally and specifically in the subgroup of GFR < 60 ml/min/1.73 m^2^ and both BMI subgroups.

## 1. Introduction

According to the 21st Malaysian Dialysis and Transplant Registry report, in the year 2013, a total of 31,637 patients received dialysis, an increase from a mere 11,842 in 2004. A staggering 61% of end-stage renal disease (ESRD) in Malaysia was reported to be caused by diabetes mellitus [1]. Chronic kidney disease (CKD) can lead to various complications and is well known to be an independent risk factor for cardiovascular disease [2]. A reduced glomerular filtration rate (GFR) to <60 ml/min/1.73 m^2^ alone is sufficient to diagnose CKD [3]. Direct assessment of GFR is measured from urinary or plasma clearance of an ideal filtration marker such as inulin or other alternative exogenous markers such as iothalamate, chromium 51 ethylenediaminetetraacetic acid (^51^Cr-EDTA), technetium-99 m diethylenetriaminepentaacetic acid (^99m^TC-DTPA), and iohexol. ^51^Cr-EDTA and ^99m^TC-DTPA are radioactive tracers that were reported in radiological studies used to obtain accurate measurement of GFR [4, 5]. However, measuring clearance with exogenous markers is complex, expensive, and difficult to do in routine clinical practice. Therefore, an accurate, convenient, and precise method to estimate GFR is important to overcome this problem.

Traditionally, serum creatinine has been used as a marker to assess kidney function. It is now an established fact that serum creatinine alone is not an accurate marker of GFR as it is dependent on muscle mass [6]. Apart from that, serum creatinine usually does not increase until GFR has decreased by 50% or more and thus many patients with normal serum creatinine may have lower GFR [7]. Therefore, a calculated GFR from creatinine-based method is recommended. In Malaysia, Cockcroft-Gault (CG) formula for estimating kidney function is still widely used. Unfortunately it has been reported to overestimate true GFR. The Modification of Diet in Renal Disease (MDRD) formula derived from MDRD study was proposed to overcome this limitation [8]. Based on the study, four-variable MDRD (4-MDRD) that consists of serum creatinine, gender, age, and ethnicity was derived and became commonly used in clinical practice and research. The 4-MDRD formula provides good GFR estimation particularly in the group of GFR <60 mL/min/1.73 m^2^ White Americans [9]. This subsequently leads to the new equation proposed for Caucasian and African-American CKD populations, known as Chronic Kidney Disease Epidemiological Collaboration (CKD-EPI) equation [10]. The development of this equation is mainly to overcome some of the limitations from MDRD equation, particularly in estimating GFR of >60 ml/min/1.73 m^2^.

Among Asian population, namely, in Chinese, Japanese, and Thais, racial coefficient has been identified and incorporated in eGFR formulas [11–14]. To date, studies comparing different methods of kidney function assessment in our unique multiethnic population are very scarce. Evaluation of these methods in the Malays as the dominant ethnic group of this country is very interesting. A good eGFR formula needs to have lower bias and limits of agreement, in addition to excellent precision and accuracy. The objective of this study is to evaluate the accuracy of creatinine-based eGFR formulas compared to the measured GFR in Malay population.

## 2. Materials and Methods

This is a cross-sectional study conducted in University Malaya Medical Centre (UMMC), Kuala Lumpur, Malaysia, and approved by UMMC ethic committee. We used power and sample size software version 3 to calculate sample size. Single mean formula was used. Under a significance level of 0.05 and power of 0.90, the estimated sample size is 46 ± 10% patients. Our study cohort involved patients presented to UMMC nephrology clinic for their regular follow-up. Volunteered participants were recruited in continuous manner. All patients older than 18 years old with stable renal function for at least 3 months prior to recruitment were eligible to participate. Patients with acute deterioration of renal function, bedridden patients, patients with malnutrition, limb amputees, patients who are less than 18 years old, and pregnant women were excluded.

### 2.1. ^51^Cr-EDTA Measurement

Measured GFR is determined by collecting blood sample from different arm 2, 2.5, 3, and 4 hours later following ^51^Cr-EDTA single injection technique. Plasma clearance of ^51^Cr-EDTA from 4 samples was obtained based on the interval above. Patient's height and weight were measured for body surface area (BSA) calculation. GFR was calculated using the slope-intercept method and normalized to BSA, which was calculated using du Bois formula. The result was then corrected using Brochner-Mortensen equation.

Volume distribution (Vd) is calculated by

(1) Vd=Standard  activity  (cpm)×  weight  of  dose×100 mlPo  (cpm) ×weight  of  standard.

- Standard activity is calculated using computer generated chromium result.
- Weight of dose is calculated from weight of syringe and dose before injection − after injection.
- Po (zero time plasma activity) is corrected by extrapolating the curve to zero time.

Slope clearance (C-slope) is calculated by

(2) $$\begin{array}{c} \frac{\text{C-slope}}{\text{slope intercept}}=\frac{0.693}{T\left(1/2\right)}\times Vd. \end{array}$$

Normalized GFR is calculated by

(3) $$\begin{array}{c} Normalized\;\;GFR=\frac{\text{C-slope}}{\text{Patient's BSA}}\times 1.73. \end{array}$$

### 2.2. Calibration for the Serum Creatinine Assay

Serum creatinine was measured on a Dimension Vista system clinical chemistry analyzer (Siemens) with an assay using a modification of the kinetic Jaffe reaction (alkaline picrate reaction). This modified technique was reported to be less susceptible than conventional methods to interference from noncreatinine Jaffe positive compounds [15]. The creatinine assay was adjusted for calibration with the isotope dilution mass spectrometry (IDMS).

### 2.3. Estimated GFR Calculations

The eGFR values were calculated by using CG, 4-MDRD, and CKD-EPI equations. 4-MDRD and CKD-EPI derived eGFR are expressed as ml/min/1.73 m^2^. Meanwhile CG equation was converted from ml/min to ml/min/1.73 m^2^ by multiplying the calculated values by 1.73 and dividing by BSA (Table 1).

### 2.4. Statistical Analysis

SPSS version 20.0 was used to calculate baseline characteristics frequency, mean, median, range, and standard deviation. Mean GFR were given with a 95% confidence interval (CI) unless indicated otherwise. *p* values < 0.05 were considered significant. Pearson's correlation coefficients (*r*) were calculated between ^51^Cr-EDTA clearance and estimated GFR by a linear correlation analysis. Pairwise comparison of the mean was performed using paired *t*-test.

Bias, precision, and accuracy within 10% and 30% of the measured GFR were determined. Bias is defined as mean difference between estimated GFR and the measured GFR (^51^Cr-EDTA). The precision of the estimates was determined as SD of the mean difference between measured GFR and eGFR. Accuracy was determined by integrating precision and bias and was calculated as the percentage of GFR estimates within 10 and 30% of the measured GFR. Moreover, a graphical analysis was carried out according to Bland and Altman plots. This was used to assess the limits of agreement between the eGFR and the measured GFR.

In our study, accuracy is the most important determinants for a good estimated GFR and it is best if further supported by lower bias, greater precision, and lower limits of agreement. However, as we understand that bias, precision and limits of agreement may be affected by the overall means and outliers; therefore the individual parameter may not reflect the best estimated GFR.

## 3. Results

A total of 51 patients were recruited with mean age of 58.7 years, where the youngest was 26 years old and the eldest was 78 years old. Majority of our patients are males representing 90.2%. The mean height and weight in our patient were 164.5 cm and 71.9 kg, respectively, with mean BMI of 26.5 kg/m^2^. Vast majority of our study patients had diabetic nephropathy (35.3%) and hypertension (19.6%) as the main cause of their CKD. Summary of patient's baseline characteristics is tabulated in Table 2.

From our cohort, mean measured GFR was 42.04 (17.70–111.10) ml/min/1.73 m^2^, while the estimated GFR based on CGBSA, 4-MDRD, and CKD-EPI formula were 40.47 (16.52–115.52), 35.90 (14.00–98.00), and 37.24 (14.00–121.00), respectively. The calculated GFR of the 4-MDRD and CKD-EPI differed significantly from measured GFR with *p* value = 0.001 and 0.005. The correlation between estimated and measured GFR is illustrated in Table 3.

Bias of CGBSA (1.573 ml/min/1.73 m^2^) was smaller than 4-MDRD (6.137 ml/min/1.73 m^2^) and CKD-EPI (4.804 ml/min/1.73 m^2^), while the precisions of the estimated GFR showed that CGBSA is more precise followed by CKD-EPI and 4-MDRD formula. However, from our cohort we found that 4-MDRD is the most accurate formula with the accuracy of 13.7 and 54.9% within 10 and 30% of measured GFR, respectively. Nevertheless, we noted that 4-MDRD formula underestimated GFR by 6.137 ml/min/1.73 m^2^; this was likely because of the outliers in this study cohort.

**Figure 1:**
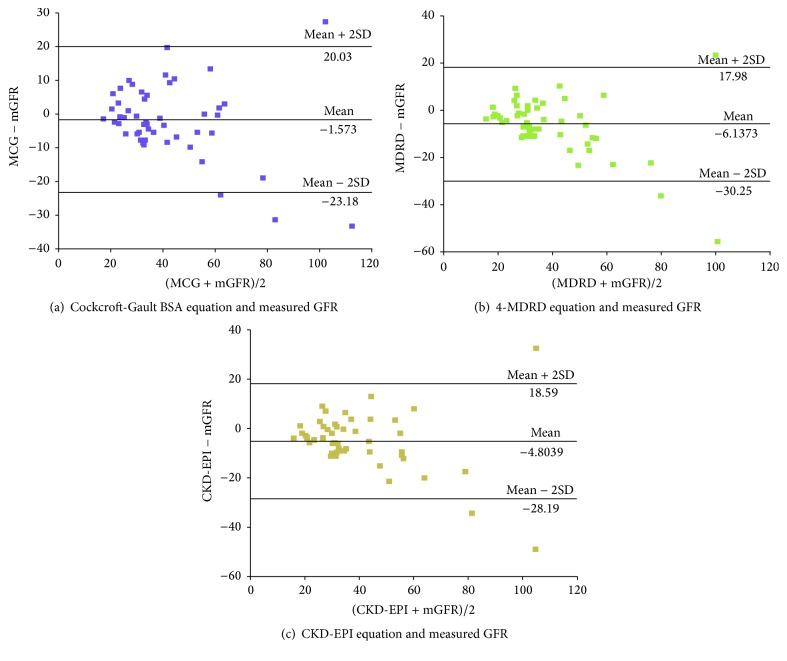
(a–c) Bland and Altman analysis of GFR estimates. In this analysis, the differences between estimated and measured GFR are plotted against the average of the estimated and measured GFR for each individual patient.

The differences between estimated and measured GFR were illustrated using a graphical technique according to Bland and Altman plot (Figures 1(a)–1(c)). These figures display the span between +2SD and −2SD of the mean difference (limits of agreement between 2 methods), which represent 95% CI. From the chart below it showed that smaller limits of agreement were found for the CGBSA (43.21 ml/min/1.73 m^2^), followed by CKD-EPI (46.78 ml/min/1.73 m^2^) and 4-MDRD (48.23 ml/min/1.73 m^2^) formula. Even though limits of agreement in 4-MDRD formula are wider, Figure 1(b) illustrated that each patient distribution is closer from one another and these wider limits of agreement can be explained by the extreme outliers (underestimated by almost 60 mls/min/1.73 m^2^) that present in this group. Thus, this make 4-MDRD formula the most accurate estimated GFR in comparison with ^51^Cr-EDTA throughout all ranges of GFR in our study cohort.

Patients were further divided into two groups according to the measured GFR: GFR < 60 ml/min/1.73 m^2^ or GFR ≥ 60 ml/min/1.73 m^2^. In subgroup GFR < 60 ml/min/1.73 m^2^, lower bias was found for CGBSA formula (0.34 ml/min/1.73 m^2^) followed by CKD-EPI (2.24 ml/min/1.73 m^2^) and 4-MDRD (2.95 ml/min/1.73 m^2^). However, better accuracy within 10% of measured GFR was found in 4-MDRD and CKD-EPI formula. In subgroup GFR ≥ 60 ml/min/1.73 m^2^, a different pattern of bias and accuracy was noted. In this subgroup, CGBSA formula was found to be better in terms of bias (9.40 ml/min/1.73 m^2^) and accuracy within 10% of measured GFR (40%), while 4-MDRD and CKD-EPI formula were noted to have higher bias, 19.22 and 15.32 ml/min/1.73 m^2^, respectively, and lower accuracy within 10% of measured GFR. Precisions of all the equations were significantly lower in the patients with GFR <60 ml/min/1.73 m^2^ (Table 4).

Assessment of eGFR formula in patients with BMI < 23 kg/m^2^ and BMI ≥ 23 kg/m^2^ was performed. In both subgroups, better accuracy within 10 and 30% of measured GFR was found in 4-MDRD formula, which was 14.3 and 50% in BMI < 23 kg/m^2^ while in subgroup BMI ≥ 23 kg/m^2^ was 16.2 and 54.0% (Table 5).

## 4. Discussions

This study investigated the performance of different creatinine-based eGFR formula in Malay population in a tertiary hospital in Malaysia. An accurate eGFR measurement is extremely important as a tool for CKD diagnosis, drug dosage preparations, and procedural preparation and subsequently to determine the efficacy of novel treatments to delay CKD progression in clinical practice. Performing labor-intensive radio-labelled GFR measurement is not practical and economical particularly in developing country like Malaysia.

It is known that racial coefficient is an important factor to determine accurate GFR [11–14, 16, 17]. In our cohort, the eGFR obtained from each formula showed significant correlation with measured GFR (^51^Cr-EDTA). However, the eGFR by 4-MDRD formula in general was found to be more accurate than the other eGFR equations in estimating GFR in our small cohort.

In the subgroup analysis of measured GFR < 60 mls/min/1.73 m^2^, our data showed that CKD-EPI and 4-MDRD formulas showed better performance pertaining to the accuracy in comparison with other estimates GFR. The results corresponded with MDRD study that was performed in White American patients, which revealed that MDRD equation showed a reliable performance in estimating GFR in CKD patients with GFR < 60 mls/min/1.73 m^2^. However, in Singaporean multiethnic study, it revealed that CKD-EPI was more accurate than the 4-MDRD in GFR < 60 ml/min/1.73 m^2^ and overestimated reference GFR when the reference GFR was ≥ 60 ml/min/1.73 m^2^ [18].

Estimating GFR in overweight and obese populations is another interesting factor to look into as weight and body size may influence the level of creatinine. In subgroup analysis of BMI ≥ 23 kg/m^2^, greater accuracy was noted in 4-MDRD formula. Similar result was noted in another local study done by National University of Malaysia (UKM) that revealed MDRD equation showed greater accuracy and precision in obese individuals [19]. Interestingly, in BMI < 23 kg/m^2^, 4-MDRD fared better as well unlike in lean population in African that showed that CG was better than MDRD and CKD-EPI formula with regard to the narrow limits of agreement [16].

## 5. Limitations of the Study

This is a small single-centre cohort of CKD patients, who are predominantly male and mainly consisted of CKD stages 3 and 4. Due to the continuous sampling method used in this study, we are unable to ensure equal distribution of patients in different arms of subgroup analysis. Thus, to further validate the more recent CKD-EPI formula, more inclusion of other stages of CKD is needed. Although this study has the above-mentioned limitations, this is the first study to be conducted in Malaysia using ^51^Cr-EDTA as reference GFR.

## 6. Conclusion

We found that 4-MDRD equation seems to be more accurate in estimating GFR in our small cohort of Malay CKD patients except in subgroup of GFR ≥ 60 mls/min/m^2^, where CGBSA was found to be better. We would like to propose further studies to look into the need for racial correction factor to improve the performance of the original 4-MDRD formula in Malay population.

## Figures and Tables

**Table 1:** Different eGFR formula according to gender.

eGFR methods	Gender	Equations
Cockcroft-Gault	Male	$\frac{\left(140\text{-}Age\right)\times mass\;\;(kg)\times 1.23}{Serum\;\;Creatinine\;\;(umol/L)}$
	Female	$\frac{\left(140\text{-}Age\right)\times mass\;\;(kg)\times 1.04}{Serum\;\;Creatinine\;\;(umol/L)}$

4-MDRD	Male	32788 × Serum Creatinine^−1.154^ × Age^−0.203^ × {1.212 if Black}
	Female	32788 × Serum Creatinine^−1.154^ × Age^−0.203^ × {1.212 if Black} × 0.742
		(Serum creatinine in umol/L)

CKD-EPI	Male	141 × min⁡(SCr/0.9,1)^−0.411^ × max⁡(SCr/0.9,1)^−1.209^ × 0.993^Age^ × {1.159 if Black}
	Female	141 × min⁡(SCr/0.7,1)^−0.329^ × max⁡(SCr/0.7,1)^−1.209^ × 0.993^Age^ × {1.159 if Black} × 1.018

Cockcroft-Gault BSA		$\frac{\text{Calculated Cockcroft-Gault}\times 1.73}{\text{BSA}}$

**Table 2:** Baseline characteristics of patients.

Characteristic (*n* = 51)	Mean ± SD (median) or *n* (%)
Male	46 (90.2)
Age (year)	58.7 ± 12.6 (61.0)
BMI (kg/m^2^)	26.5 ± 4.6 (25.5)
Plasma creatinine (umol/l)	192.5 ± 66.7 (190.0)
Plasma urea nitrogen (mmol/l)	9.8 ± 3.5 (9.4)
Plasma albumin (g/l)	37.9 ± 3.0 (38.0)
Measured GFR (ml/min/1.73m^2^)	42.04 ± 22.5 (35.1)
Causes of CKD	
Diabetic nephropathy	18 (35.3)
Hypertension	10 (19.6)
Nondiabetic glomerulopathy	4 (7.8)
Renal calculi/nephrocalcinosis	4 (7.8)
Other causes	10 (19.7)
Unknown	5 (9.8)
CKD stages	
1	2 (3.9)
2	8 (15.7)
3	26 (51.0)
4	15 (29.4)
Medical history	
Diabetes mellitus	33 (64.7)
Hypertension	46 (90.2)
Medications	
Diuretics	14 (27.5)
Antihypertensive	48 (94.1)
OHA/insulin	32 (62.7)
Statin	41 (80.4)
Smoking status	
Current smoker	6 (11.8)
Ex-smoker	21 (41.2)
Nonsmoker	24 (47.1)

**Table 3:** Correlation coefficient (*r*), mean, bias, precision, and accuracy for CGBSA, 4-MDRD, and CKD-EPI formula.

	Correlation coefficient (*r*)	Mean GFR	Range (IQR)		*p* value	Mean difference (bias)	SD of mean bias (precision)	Accuracy within	
			Lower	Upper				10%	30%
Measured GFR		42.039	17.70	111.10					
CGBSA	0.877^*∗*^	40.467	16.52	115.52	0.303	−1.573	10.802	9.8	47.1
4-MDRD	0.848^*∗*^	35.902	14.00	98.00	0.001	−6.137	12.058	13.7	54.9
CKD-EPI	0.854^*∗*^	37.235	14.00	121.00	0.005	−4.804	11.697	13.7	49.0

^*∗*^Significantly correlating with *p* < 0.001.

(Bias: mean difference of estimated GFR and measured GFR; accuracy: *n* percentage of GFR estimates within *n*% of measured GFR; IQR: interquartile range).

**Table 4:** Mean, bias, precision, and accuracy of GFR estimates within two GFR subgroups.

Variable	GFR < 60 ml/min/1.73 m^2^ (*n* = 41)	GFR ≥ 60 ml/min/1.73 m^2^ (*n* = 10)
*GFR* (ml/min/1.73 m^2^)		
Measured	33.19 ± 10.39	78.32 ± 22.61
CGBSA	33.53 ± 10.79^*∗*^	68.92 ± 21.10^*∗∗*^
4-MDRD	30.24 ± 10.27^*∗*^	59.10 ± 21.35^*∗∗*^
CKD-EPI	30.95 ± 11.14^*∗*^	63.00 ± 23.49^*∗∗*^
*Median bias*		
CGBSA	−0.99 (−9.6, 19.81)	−9.80 (−33.00, 27.35)
4-MDRD	−2.60 (−17.10, 10.20)	−19.70 (−55.80, 23.60)
CKD-EPI	−1.80 (−15.10, 13.20)	−14.70 (−48.80, 32.60)
*Mean difference*		
CGBSA	0.34 ± 7.04	−9.40 ± 18.53
4-MDRD	−2.95 ± 6.13	19.22 ± 20.11
CKD-EPI	−2.24 ± 6.22	−15.32 ± 20.86
*Accuracy within 10*%		
CGBSA	24.4	40.0
4-MDRD	31.7	20.0
CKD-EPI	31.7	10.0
*Accuracy within 30*%		
CGBSA	63.4	70.0
4-MDRD	65.9	80.0
CKD-EPI	65.9	80.0

^*∗*^Mean CGBSA GFR versus measured GFR *p* = 0.761, mean 4-MDRD GFR versus measured GFR *p* = 0.004, and mean CKD-EPI GFR versus measured GFR *p* = 0.026.

^*∗∗*^Mean CGBSA GFR versus measured GFR *p* = 0.143, mean 4-MDRD GFR versus measured GFR *p* = 0.014, and mean CKD-EPI GFR versus measured GFR *p* = 0.045.

**Table 5:** Mean, bias, precision, and accuracy of GFR estimates within two BMI subgroups.

Variable	BMI < 23 kg/m^2^ (*n* = 14)	BMI ≥ 23 kg/m^2^ (*n* = 37)
*GFR* (ml/min/1.73 m^2^)		
Measured	43.19 ± 19.44	41.61 ± 23.78
CGBSA	41.80 ± 23.96^*∗*^	39.94 ± 17.67^*∗∗*^
4-MDRD	43.14 ± 23.28^*∗*^	33.16 ± 13.90^*∗∗*^
CKD-EPI	44.57 ± 25.73^*∗*^	34.46 ± 15.40^*∗∗*^
*Median bias*		
CGBSA	−1.34 (−23.8, 27.35)	−1.66 (−33.07, 19.81)
4-MDRD	0.04 (−22.8, 23.6)	−8.44 (−55.80, 6.10)
CKD-EPI	1.39 (−19.8, 32.6)	−7.15 (−48.80, 7.10)
*Mean difference*		
CGBSA	−1.34 ± 11.54	−1.66 ± 10.68
4-MDRD	−0.04 ± 11.06	8.44 ± 11.74
CKD-EPI	1.39 ± 12.31	−7.15 ± 10.71
*Accuracy within 10*%		
CGBSA	7.0	8.1
4-MDRD	14.3	16.2
CKD-EPI	14.3	10.8
*Accuracy within 30*%		
CGBSA	50.0	48.6
4-MDRD	50.0	54
CKD-EPI	42.9	54.1

^*∗*^Mean CGBSA GFR versus measured GFR *p* = 0.672, mean 4-MDRD GFR versus measured GFR *p* = 0.989, and mean CKD-EPI GFR versus measured GFR *p* = 0.680.

^*∗∗*^Mean CGBSA GFR versus measured GFR *p* = 0.350, mean 4-MDRD GFR versus measured GFR *p* < 0.001, and mean CKD-EPI GFR versus measured GFR *p* < 0.001.
